# The impact of incidental anxiety on the neural signature of mentalizing

**DOI:** 10.1162/imag_a_00096

**Published:** 2024-02-22

**Authors:** Li-Ang Chang, Jan B. Engelmann

**Affiliations:** Center for Research in Experimental Economics and political Decision Making (CREED), Amsterdam School of Economics, University of Amsterdam, Amsterdam, The Netherlands; Behavioral Economics, The Tinbergen Institute, Amsterdam, The Netherlands

**Keywords:** anxiety, threat, mentalizing, temporoparietal junction, connectivity, false-belief task

## Abstract

While the effects of anxiety on various cognitive processes, including memory, attention, and learning, have been relatively well documented, the neurobiological effects of anxiety on social cognitive processes remain largely unknown. We address this gap using threat-of-shock to induce incidental anxiety while participants performed two false-belief tasks, a standard and an economic-games version. During belief formation and belief inferences, regions in a canonical social cognition network showed activation reflecting mentalizing, including the temporoparietal junction (TPJ), precuneus, and dorsomedial prefrontal cortex (dmPFC). At the same time, we found threat-related suppression of social cognition regions during belief inferences. A conjunction analysis confirmed that a network of regions was simultaneously engaged during mentalizing and suppressed by anxiety: bilateral TPJ, bilateral inferior frontal gyrus (IFG), and putamen. We examined how threat impacted the connectivity between these seed regions and its targets. During belief formation, we found that threat suppressed the connectivity between the precuneus and two key mentalizing nodes, the dmPFC and right TPJ. Moreover, during belief inferences, threat specifically suppressed belief-based connectivity between putamen and its targets in intraparietal sulcus (IPS) and dlPFC. Dispositional distress significantly modulated threat-related suppression of connectivity between the left TPJ and left IPS. Our results indicate that social cognitive processes rely on support from other large-scale networks, such as the reward and attentional systems, and that these network interactions are disrupted under incidental and dispositional anxiety.

## 1 Introduction

Because much of human everyday behavior occurs in the presence of some underlying background emotion, it is becoming increasingly important to understand how incidental emotions influence cognitive processes that support behavior. Anxiety is an emotion that is particularly prevalent for human behavior (Bandelow et al., 2015), as its presence signals the occurrence of negative events that can be crucial for survival. Recent research has begun to investigate the effects of such incidental anxiety on cognitive processes, including attention (Bar-Haim et al., 2007; Bradley et al., 2010; Cisler & Koster, 2010; MacLeod & Mathews, 1988), memory (Andreotti et al., 2011; Balderston et al., 2017; Bolton & Robinson, 2017; Vytal et al., 2013), and learning (Becker et al., 2019; Berghorst et al., 2013; Browning et al., 2015; Cavanagh et al., 2019; DeVido et al., 2009; Grupe, 2017; Robinson et al., 2013; Safra et al., 2018; Stevens et al., 2014; Ting et al., 2022). Despite this recent surge in the interest concerning the effects of incidental anxiety on cognitive processes and its neural correlates, relatively little is known about its impact on arguably one of the most important cognitive processes for human interaction, namely social cognitions such as mentalizing. In the current experiment, we aimed at addressing this gap in the literature and investigated how incidental anxiety impacts the underlying neural circuitry that supports mentalizing.

Mentalizing (also commonly referred to as Theory of Mind) is defined as the ability to attribute mental states to others, such as beliefs, intentions, and desires, and thereby enables reasoning about others’ mental states (Frith & Frith, 2006; Premack & Woodruff, 1978). As such, mentalizing supports human interaction in important ways. Previous work has begun to delineate the effects of anxiety on social cognitions, such as mentalizing. Clinical work suggests that anxiety disorders are associated with diminished mentalizing abilities. Compared to healthy controls, patients with social anxiety disorder (SAD) have been shown to perform relatively poorer on the reading the mind in the eyes task (RMET) (Hezel & McNally, 2014), as well as related tasks including the Movie for the Assessment of Cognition (MASC) (Dziobek et al., 2006; Washburn et al., 2016). Recently, a number of meta-analyses have further substantiated the link between pathological anxiety and a diminished ability to understand and reason about others’ perspective. Not only has social anxiety been found to negatively affect social cognitive capacity in children and adolescents (Pearcey et al., 2021), but this association also extends to other anxiety disorders and older patients. Sloover et al. (2022) observed a pattern of reduced mentalizing abilities across various patient groups, including those with anxiety disorders, obsessive-compulsive and related disorders, as well as trauma and stress-related disorders. In a more recent meta-analysis, Chevalier et al. (2023) showed a general and negative association between anxiety and mentalizing performance across multiple tasks and questionnaires, developmental stages, and anxiety disorders. Of note, the sometimes contradictory findings and the relatively small global effect observed in recent meta-analyses suggest that the reduced mentalizing capacities, while consistently demonstrated in clinical and subclinical samples and across developmental periods, likely do not reflect a general deficit in anxiety disorders. Instead, the reductions in mentalization abilities observed in anxious individuals may be situation dependent, such that they surface mostly in stressful contexts (Chevalier et al., 2023).

The notion that anxiety suppresses social cognition is further supported by studies that induce incidental anxiety in healthy subjects, demonstrating causal effects of anxiety that move beyond cross-sectional and correlational experimental designs. An initial set of experiments by Todd et al. (2015) induced incidental anxiety via an autobiographical recall task before healthy participants completed spatial and conceptual perspective taking tasks. Results indicate that anxious participants exhibited greater egocentrism compared with participants experiencing neutral and other negative emotions, such as anger and disgust. These results were replicated across a number of different spatial and conceptual perspective taking tasks and were specific for social settings (Todd & Simpson, 2016; Todd et al., 2015).

Despite these initial behavioral findings on the effects of anxiety on mentalizing, relatively little is known about how incidental anxiety affects the underlying neurobiology that supports social cognition. The neurobiological basis of both mentalizing and anxiety have been delineated separately by extensive prior neuroimaging work in social and affective neuroscience. Several meta-analyses agree on a core neural network that supports mentalizing across different tasks, consisting of bilateral temporoparietal junction (TPJ), medial prefrontal cortex (mPFC), superior temporal sulcus (STS), temporal pole (TP), and precuneus (sometimes including posterior cingulate cortex, PCC) (Amodio & Frith, 2006; Bzdok et al., 2012; Decety & Lamm, 2007; Mar, 2011; Mitchell, 2009; Molenberghs et al., 2016; Schurz et al., 2014; Van Overwalle, 2009). Paralelly, a recent fMRI meta-analysis that summarized the past two decades of neuroimaging research on anxiety identified a “core anxiety” network whose regions include the amygdala, the bed nucleus of the stria terminalis (BST), periaqueductal gray, midcingulate cortex, and anterior insula (Chavanne & Robinson, 2021). Of importance for the current study is the finding that these regions are involved in *both* clinical anxiety and pre-clinical studies that employ anxiety induction, suggesting that emotional challenges recruit regions within this network regardless of pre-existing symptomatology related to anxiety (Chavanne & Robinson, 2021).

While relatively little is known about the neurobiological effects of incidental anxiety on mentalizing, a prior neuroimaging study investigated the impact of induced stress on participant’s performance in the reading the mind in the eyes test (RMET, Nolte et al., 2013). The fMRI results showed that attachment-related stress induction, which asked subjects to think about stressful circumstances relevant to interpersonal relationships, suppressed several mentalizing areas, particularly in the left posterior superior temporal sulcus (STS), left temporoparietal junction (LTPJ), and left inferior frontal gyrus (IFG). While the STS and TPJ are part of a core social cognition network, the IFG is commonly considered a cognitive control region that can also be viewed as part of an extended social cognition network (Arioli et al., 2021). The role of the IFG in social cognition is reflected by results showing diminished IFG activation while children with Autism Spectrum Disorder (ASD) observe and imitate emotional expressions (Dapretto et al., 2005), and negative correlations between left IFG lesions and RMET performance in traumatic brain injury patients (Dal Monte et al., 2014). More recently, Engelmann, Meyer et al., 2019 identified the ventrolateral PFC (including IFG) as part of a network of social cognition regions that also include pSTS, dmPFC, and amygdala, whose connectivity strength with TPJ is specifically associated with trust game investments. At the same time, this region showed threat-related suppression during trust and non-social investment decisions. Jointly, evidence from the studies reviewed above suggests that stressors can suppress the activation of neural circuitry associated with social cognition.

On the other hand, patients with social anxiety disorder show hyperactivation in brain regions associated with mentalizing compared to healthy controls during multiple tasks. A common finding from studies with SAD patients is hyperactivation in midline structures, such as the mPFC and PCC during a number of self-referential tasks including self-referential working memory (Yoon et al., 2016) and emotion regulation tasks (Gaebler et al., 2014), as well as during resting state (e.g., Rabany et al., 2017; see review by Yoon et al., 2019). Additionally, the hyperactivation found in SAD patients is not limited to midline structures, but can also involve the TPJ (Boehme et al., 2015; Gaebler et al., 2014; Yoon et al., 2016). The studies reviewed above primarily focus on populations with chronically increased levels of anxiety such as dispositional anxiety and anxiety disorders. Thus, it remains unclear whether incidental anxiety, which arises from environmental triggers, affects the mentalizing network in similar ways in healthy populations. Results of Chavanne and Robinson (2021) showing significant overlap in anxiety-related activation in clinical samples and after anxiety induction in healthy controls tentatively suggest similar neural effects for clinical and induced anxiety. We test here whether this extends to the neural correlates of the effects of incidental anxiety on social cognition.

Social cognitive processes also crucially support social interaction during economic games. Therefore, if anxiety interferes with these social cognitive processes during economic game interaction, it should lead to distorted economic behavior. FeldmanHall et al. (2015) applied the cold pressor test (CPT) to induce stress before participants performed a trust game. Results showed diminished trust compared to a non-social control condition, specifically after stress induction. These results were confirmed and extended by a more recent fMRI experiment in which participants played a trust game under conditions of incidental anxiety, induced via threat-of-shock (Engelmann, Meyer et al., 2019). Behavioral results confirmed the earlier findings, showing decreased levels of trust under conditions of incidental anxiety compared to safety. At the neural level, anxiety was associated with supressed left TPJ activity and suppressed functional coupling between left TPJ and amygdala. Moreover, while the functional connectivity between the left TPJ and its targets in pSTS (posterior superior temporal sulcus), dmPFC, amd vmPFC was associated with the transferred amount in the trust game (but not in the non-social control condition), anxiety speficifcally disrupted this brain-behaviour relationship between left TPJ and pSTS. Jointly, these results indicate that interpersonal behaviors, such as trust, can be disrupted by anxiety. Moreover, the finding that the disruption of this behavior correlates with specific suppression of activity and connectivity between regions involved in mentalizing further suggests that these effects of anxiety may be driven primarily by a disruption of social cognitive processes.

In the current study, we aim to provide direct evidence for the disruption of mentalizing in the presence of incidental anxiety and delineate the effects of anxiety on the activity and connectivity within the social cognition network. Specifically, we ask whether and how anxiety influences the ability to mentalize and to what degree anxiety disrupts the social cognition network that supports mentalizing. To accomplish this, we asked our subjects to complete two different false-belief tasks for which they first read vignettes about life stories (the standard FBT), or about interactions in economic games (the economic-game FBT). Our participants did this either in the presence of threat of shock, or in its absence. We inspected the effect of threat of shock on mentalizing-related activation and connectivity during two periods: (1) a vignette reading period, in which participants were required to form beliefs about agents in vignettes, and (2) during a question period, in which participants were asked to answer incentivized questions about the agents’ beliefs and intentions. We expected impaired behavioral performance and suppressed activity and connectivity during threat in mentalizing regions. More specifically, we expected anxiety to suppress activity in key regions of the social cognition network. Given our previous results (Engelmann, Meyer et al., 2019) demonstrating threat-related suppression of activity during trust decisions, we expected similar threat-related suppression during mentalizing particularly in the TPJ, but also in additional social cognition regions including the dmPFC, precuneus, and inferior frontal gyrus. We expected an extended social cognition network to be affected by threat in the current experiment because belief formation and belief inferences during vignettes likely reflect more general mentalizing processes compared to trust decisions, which involve a more targeted form of mentalizing that is highly context specific. We also expected functional connectivity to be disrupted in the presence of anxiety, such that connectivity between key regions in the social cognition network is relatively suppressed under conditions of anxiety. Finally, we conducted exploratory analyses investigating the modulatory role of dispositional distress in connectivity strength.

## 2 Materials and Methods

### 2.1 Subjects

Thirty-nine volunteers participated in the current experiment (18 males, aged 18 - 33, mean (SD) = 22.51 (4.03) years). Two subjects were excluded from data analyses, one due to excessive head motion (>2 voxels (6 mm) in translation and rotation) and one due to subthreshold task performance (mean accuracy <3 standard deviation of the sample mean). The final dataset for the main analysis therefore included 37 subjects. For analyses of individual differences, one further subject needed to be excluded due to technical issues with the online questionnaire that led to data loss. Participants were recruited from the subject pool of the Behavioral Science Lab at the University of Amsterdam (https://www.lab.uva.nl/lab/home) and provided informed consent to procedures that were approved by the Ethics Review Board of the Faculty of Social and Behavioral Sciences of the University of Amsterdam (ERB# 2018-EXT-9293).

### 2.2 Timeline of procedures

In the first part of the experiment, participants were invited to fill in an online prescreening questionnaire and a battery of personality measures via Qualtrics. The questionnaire battery was adapted from (Engelmann, Schmid, et al., 2019) and is outlined in detail in the factor analysis section of the Supplementary Materials (Fig. S6, Table S3). Subjects received 14 Euros for completing this part of the study. In the second part of the experiment, subjects were invited to the Behavioral Science Lab at the University of Amsterdam. They were asked to read the instructions thoroughly, after which their questions were discussed with the experimenter, followed by a quiz that assessed their understanding of the task, and, in particular, the economic games (Trust and Ultimatum Games). Participants then completed 12 practice trials outside the scanner, which were repeated until performance was at least 66% (only 3 subjects required repetition and all of them passed the criteria on their second try). After entering the scanner, participants first underwent shock calibration and were then familiarized with the MRI compatible button box. To control for possible (de)sensitization to the electric shocks, a second shock calibration was conducted at the halfway point of the experiment (after completion of run two out of four). After scanning, subjects filled out an exit questionnaire which included additional manipulation checks (emotion ratings) and personality measurements (Honesty-Humility scale of the HEXACO-PI-R (Lee & Ashton, 2010) and the Dark Factor of Personality (Moshagen et al., 2018). Note that emotion ratings were obtained after completion of the main experiment to not interrupt the flow of the experiment and to avoid task switching. Emotion ratings are therefore based on a recent memory and reflect the average perceived emotion in threat and safe blocks. Subjects were paid an average of 32.32 Euros for their participation via the online reimbursement system of the University of Amsterdam.

### 2.3 False-belief task description

We employed a vignette-based false-belief task to investigate the role of anxiety in social cognition, focusing in particular on belief formation, which occurred during the vignette period, and belief inferences, which occurred during the question period. Specifically, we combined well-established and slightly modified (to improve readability) vignettes from prior research (adapted from Bruneau et al., 2012; Saxe & Kanwisher, 2003), with a set of novel vignettes that outline interactions in well-established economic games. For a detailed analysis of these stimuli and their neural correlates, see our previous paper (Chang et al., 2023). To complete the task, participants were asked to first read the vignettes and subsequently answer incentivized questions about the beliefs of protagonists and outcomes in different experimental conditions.

**Fig. 1.**
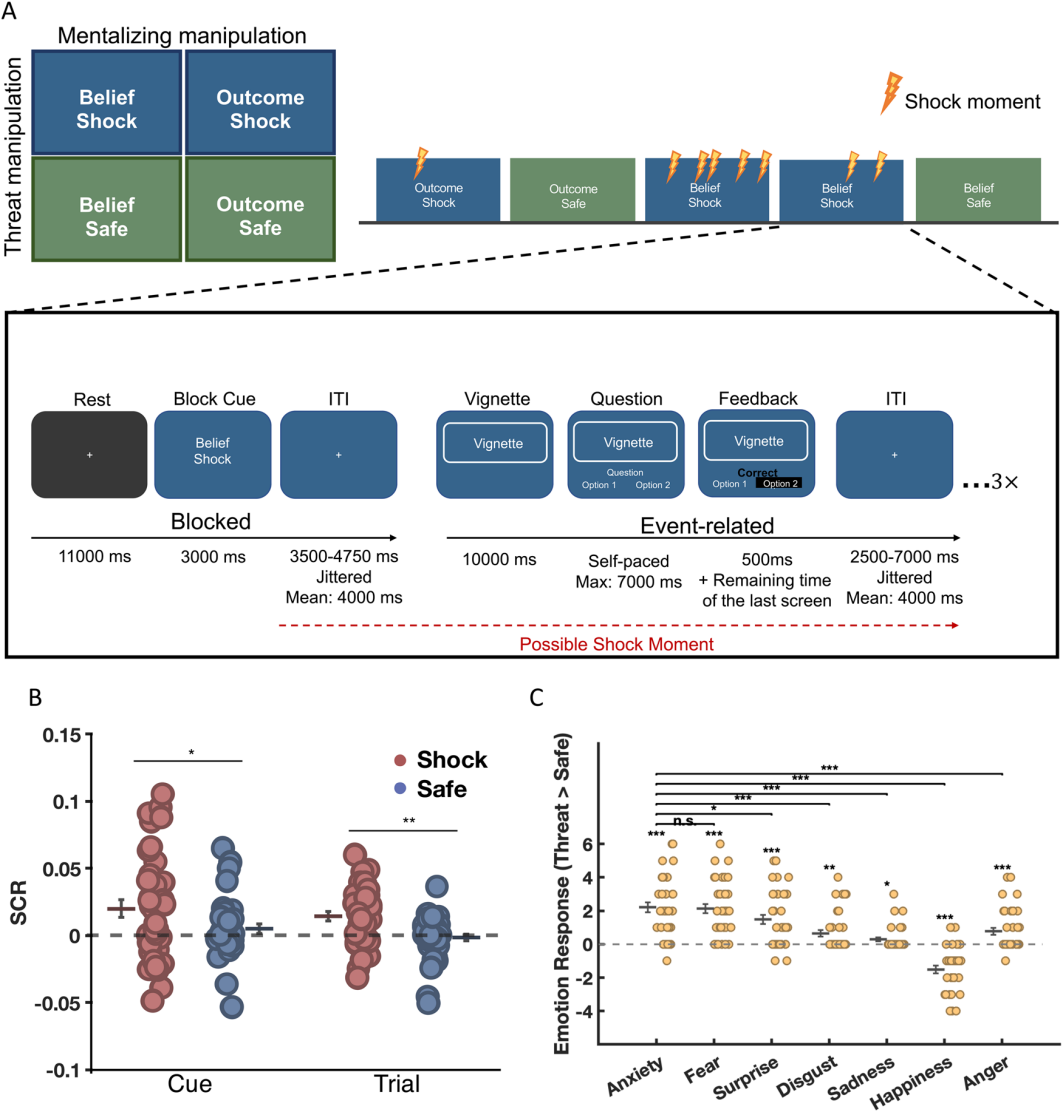
Experimental conditions, trial sequence, timing and manipulation checks. (A) The left subfigure illustrates the 2 x 2 factorial design with the crossed mentalizing and threat conditions. The right subfigure shows the implementation of the hybrid fMRI design in which the Threat and Belief factors were consistent for the duration of a block, as indicated by the block cue, and vignettes, questions, and feedback were presented in an event-related fashion. The feedback provided information on correct, incorrect, or slow responses, where only the former was paid at a 20c piece rate. The example shows the Econ-Belief-Threat condition. (Note: the background color associated with the shock condition was counterbalanced across subjects, but remained the same for each subject from practice until the end of the experiment). Manipulation checks show significant threat effects on (B) skin conductance responses and (C) self-reported emotion. (B) Significantly larger skin conductance responses were observed in the threat (red dots) compared to the safe (blue dots) condition during two phases of the experiment: the cue period and throughout the trial period. Dots represent each individual’s estimated parameter extracted from the SCR GLM (see SCR recording and analysis approach) during the cue period and the trial period after cue offset. (C) Subjects reported significantly higher levels of fear, anxiety, and surprise in threat compared to safe blocks, while reporting greater happiness in safe compared to threat blocks. In addition, the increase in self-reported anxiety in threat relative to safe blocks is significantly greater compared to all other emotions, except for fear and surprise. In both subplots (B and C), the horizontal line represents the mean, and the black error bar represents the standard error of the mean (SEM). Each dot represents an individual participant. *p* < 0.05*; *p* < 0.01**; *p* < 0.001***.

As shown in Figure 1A the experiment employed a within-subjects factorial design with the factors Domain (economic games, life stories), Belief (belief, outcome), and Threat (threat, safety). This design allowed us to test the effects of anxiety, manipulated via the well-established threat of shock procedure (see Threat of Shock procedure), on mentalizing-related neural activations (reflected via the contrast belief - outcome) in economic games and life stories. To give an example of the stimuli, in the Life Story-Outcome condition, the subjects read about events that happen to another person. They were asked to answer questions about an objective description of the consequence of the event. In the Life Story-Belief condition, the subjects were asked to infer the beliefs or intentions of the protagonist in the scenario. Economic game stimuli, on the other hand, were based on hypothetical interactions between different partners in well-established economic games, including the Trust Game (TG, Engelmann, 2010) and the Ultimate Game (UG, Thaler, 1988), and therefore required a well-founded understanding of the game setup and payout conditions to correctly answer the question. In the Economic Game-Outcome condition, the subjects were asked to calculate the payoff for one of the interaction partners based on the rules of the economic game that were outlined in detail and subsequently quizzed during the instructions. In the Economic Game-Belief condition, the subjects were required to infer the (false) beliefs of one of the interaction partners during either a Trust or Ultimatum Game interaction. The following provides an example of a trust game vignette in which participants were required to infer the false belief of one of the interaction partners (all vignettes included in the current experiment are available here https://osf.io/mdjfz?view_only=face48878dd144848d26f1c7d3c47d31):

Vignette: Eline and Kostas play a Trust Game. Each of them gets 15 Euros. Eline is the investor and sends all her money to Kostas. Kostas is confused and thinks that now they both have 60 Euros, so he sends back nothing. Eline hides her face in her hands.
Question: Eline probably thinks that Kostas: is greedy (correct) / is confused (incorrect).

Participants were informed in detailed instructions that answers were incentivized at a piece-rate of 20 cents for each correct answer, leading to additional earnings of up to €19.20 for answering all 96 questions correctly. Final payment for participation in this experiment was thus a maximum of 33.20 Euros (€19.20 for correct answers plus €14 for questionnaire completion). It was stressed in the instructions that answering correctly and on time on each trial was in the participants’ best interest in terms of the monetary payoff, but that task performance cannot influence the number (or intensity) of the electric shocks. Morevoer, participants did not receive any feedback throughout the experiment regarding their earnings to avoid reward history and associated motivational and learning effects. They were informed of their additional winnings after completion of scanning.

### 2.4 Experimental design and trial timing

We used a hybrid fMRI design that included a blocked component, namely the presence and absence of anxiety induced via threat of shock, and event-related components, namely belief formation during the vignette and belief inferences during the question periods. A specific advantage of this fMRI design is that it allows us to induce anxiety for a prolonged period of time. Furthermore, this approach reduces task switching demands (see Engelmann, Mulckhuyse et al., 2019; Ting et al., 2022). At the beginning of each block, participants were informed of the current condition via a block cue that indicated whether vignettes were presented in the economic games or life stories domain, whether questions would assess beliefs or outcomes, and whether shocks would be administered at unpredictable time points throughout the next block or not. The Threat condition was additionally communicated to participants via a constant background color that remained the same for the period of each block, such that for about half the participants a green background color was associated with threat blocks, while a blue background color was associated with safety. For the remaining subjects, this association was reversed. Participants learned about the color-threat association in the instructions and during the practice task outside the scanner. Figure 1A illustrates the sequence and timing of an example trial. Each block started with a block cue that was shown for 3000 ms and provided three pieces of information to the subjects, namely Vignette and Belief Domain, and the current Threat condition. The example in the figure shows an Econ-Belief-Shock condition, indicating that the three vignettes in the current block concern economic game scenarios, and subjects need to focus on inferring character’s beliefs, with the possibility of receiving an unpredictable number of shocks at unpredictable time points throughout the block. The block cue is then followed by a fixation cross presented for a jittered duration (mean = 4000 ms). Thereafter, subjects were asked to read a vignette, for which they were given 10,000 ms. The vignette display was followed by a question period which gave participants a maximum of 7000 ms to answer the question by choosing from two options, one correct and one incorrect option. The position of the correct option was randomized across trials. To ensure that subjects paid attention continuously throughout the experiment, correct answers within the 7000 ms-limit yielded a piece-rate payment of 20 cents that was added to participants’ cumulative earnings. The question period terminated when participants provided their answer via button press, or after the maximum allotted time of 7000 ms and was followed by feedback about their performance (shown for 500 ms plus the remaining time from the question period, that is, 7000 ms – RT, see Fig. 1A) indicating whether participants were correct, wrong, or answered too slowly. Note that our procedure prevented participants from accelerating progress on the task by responding faster, because it ensured that threat and safe blocks maintained the same average length. Participants were made aware in the instructions that they could not speed up responding in threat blocks to avoid being shocked, and that their responses on the task in no way influenced the amount, intensity, and timing of electrical shocks. Participants also did not receive any feedback about accumulated earnings throughout the experiment to avoid motivational and learning effects. Each trial was followed by an intertrial interval showing a fixation cross for a jittered period (mean = 4000 ms). The experiment consisted of a total of 96 trials distributed across 4 runs. Each run contained eight blocks, within which three trials were presented successively within the same condition.

### 2.5 Anxiety induction using threat of shock

Anticipatory anxiety was induced via the well-established threat-of-shock procedure (Engelmann, Meyer et al., 2019; Grillon et al., 2004; Hein, Engelmann, Vollberg, Tobler 2016; Ting et al., 2022), which administers unpredictable and mildly painful electric shocks in the Threat condition. Note that the anticipatory anxiety induced via this procedure translates to incidental, or task-independent, anxiety when used as an emotional context within which the current false belief tasks were presented. To induce anticipatory anxiety, the carpometacarpal joint of the participants’ non-dominant hand (always the left hand in the current study) was connected to a DS5 Isolated Bipolar Constant Current Stimulator (Digitimer Ltd.) through MRI compatible electrodes and wires. A DS5 stimulator generated stable electric shocks with a fixed maximum input of 5 V, a maximum output current of 25 mA, and a fixed shock duration of 50 ms across all subjects. The specific intensity of the electric shock was customized to each subject’s pain intensity threshold using a well-established and automated calibration procedure (e.g., Ting et al., 2022). During calibration, subjects were instructed to press a button to trigger the electrical shock themselves. After experiencing the shock, they were asked to rate how painful their experience of the electric shock was via a visual analogue scale (VAS) that ranged from 0 (no pain) to 10 (extreme pain). The automated procedure used a staircase algorithm in which ratings below 7 led to an increase of the shock amplitude by 10% of the maximum output intensity (5 V, 25 mA, duration 50 ms), ratings between 7 and 9 led to no change, while ratings of 10 led to a 10% intensity decrease. The calibration procedure terminated once three consecutive ratings of 7 to 9 were provided by each participant. If the subject provided only ratings below 7, the calibration would terminate after reaching the maximum output intensity (this occurred in 7 participants after the first calibration). If participants’ answers were highly inconsistent, the calibration terminated after 20 trials and the value of the final trial was used as shock amplitude (this did not occur for any participants). As noted in the timeline of the procedure section, the first calibration was conducted before the first run, and the second calibration was conducted at the halfway point of the fMRI experiment (after completion of the second run) to control for (de)sensitization effects. We did not find any evidence for participants purposefully selecting minimal stimulation intensities and we control for desensitization effects (Fig. S1). These results suggest that our threat induction procedures worked well.

Electrical stimulation procedures throughout the fMRI (and pilot) experiments were adapted from our prior studies (Engelmann, Meyer et al., 2019; Ting et al., 2022). Specifically, to maximize unpredictability of stimulation time points to subjects and thus to generate a robust anxiety effect (Schmitz & Grillon, 2012), both the number of shocks and the time points of electric shocks were pseudo-randomized for each threat block. Electrical shocks could be administered any time (but were kept at least 1000 ms apart) in the period spanning from the offset of the block cue until completion of the last trial in a given threat block. Possible time points of shocks were drawn from a random normal distribution throughout this time window. The number of shocks per block in each condition were pseudo-randomized for each condition within each run by selecting from a gamma function (with the values 1, 2, 2, 5) via random draw without replacement. This ensured that participants experienced the same number of shocks in each Threat condition (across the different Domain and Belief conditions). Finally, neither participants’ response speed nor their accuracy on the task could influence the intensity or the frequency of getting shocked, which we explicitly told our participants in the instructions. Jointly, these procedures, including the threat block cue, the unpredictable amount and time points of shock administration, and the sustained period throughout which electrical stimulation was possible, have successfully induced prolonged anxiety in our previous studies (Engelmann, Meyer et al., 2019; Ting et al., 2022).

### 2.6 Behavioral modeling approach

The goal of the behavioral analysis was to identify the effects of anxiety on performance and response times in the false belief task (FBT). To analyze the choice data, we conducted logistic regressions on trial-by-trial data that were implemented in the context of a generalized linear mixed-effects model (GLME) using a binomial link function. Two separate models included (1) binary responses on each trial (correct/incorrect) and (2) log reaction time as dependent variables, as well as Threat and Belief condition as fixed effects predictor variables (see Tables S1 - S3 for regression equations with all fixed and random effects). Task Domain, that is, whether participants performed the standard or economic games FBT, was not the focus of the current analyses and therefore entered as regressor of no interest in the current analyses (the Task Domain factor is discussed in more detail in a prior analysis of this data set that focuses on performance in the economic-games vs. standard false belief tasks, see Chang et al., 2023). Models were estimated via the mixed function of the AFEX package in R (Singmann et al., 2016) that relies on the lme4 package. We report results from models with the maximum possible random-effects structure (Barr, 2013). For reaction times, linear regressions using a full model structure with random slopes for the Belief factor, in addition to subjectwise random intercepts, were employed. For accuracy, logistic regressions were employed. Including all random slopes led to overfitting, requiring us to remove random slopes such that all final models include a subjectwise random intercept and random slope for Task Domain.

### 2.7 SCR recording and analysis approach

To ensure the success of the emotional manipulation, we measured participants’ skin conductance reponses (SCR). SCR data were measured using the BrainAmp ExG MR amplifier (Brain Vision, Morrisville, NC, USA) with two MR-compatible finger electrodes attached to the index and middle fingers of the participants’ nondominant left hand after conductance gel was applied. SCR data were sampled at 5000 Hz, and SCR recordings were done on a per-run basis to reduce low-frequency drift.

SCR data were preprocessed and analyzed using a general linear convolution model with a canonical skin conductance response function implemented in the software PsPM (PsychoPhysiological Modelling, Bach et al., 2009). All signals were first filtered using a bidirectional Butterworth band pass filter with cutoff frequencies of 0.0159 Hz and 5 Hz, down-sampled from 5000 Hz to 10 Hz, and subsequently z-transformed. A general linear model (GLM) was performed to estimate the threat of shock effect (threat vs. safe) during block cue presentation, as well as during the trial period. Our GLM for SCR analysis included a total of five event-related regressors, namely shock cue, safe cue, shock trial, safe trial, and the actual shock moments. All events were convolved by a canonical SCR function with a time derivative (Bach, 2014). Importantly, we also added a regressor in the model to take the shock moment *per se* into account, which enabled us to capture the affective component of the SCR signal during threat trials, which is prolonged and independent of the actual shocks that could occur at any time point throughout a given trial.

### 2.8 Functional magnetic resonance imaging

#### 2.8.1 FMRI data acquisition

FMRI data were collected using a 3.0 Tesla Philips Achieva TX MRI scanner (Philips Medical Systems, Best, The Netherlands) using a 32-channel head coil. T1-weighted structural images were acquired (1 × 1 × 1 mm voxel size resolution of 220 slices, slice encoding direction: FH axial ascending, without slice gap, TR = 8.2 ms, TE = 3.7 ms. flip angle = 8°). Functional images were acquired using a T2*-weighted gradient-echo, echo-planar pulse sequence (3.0 mm slice thickness, interslice gap = 0.3 mm, 36 ascending slices, TR = 2000 ms, TE = 28 ms, Flip angle = 76.1°, and with 240 mm field of view). In addition, to correct EPIs for signal distortion, we also conducted a field-map scan at the half-way point of the experiment for which we used a phase-difference (B0) scan (2.0 × 2.0 × 2.0 mm voxel size resolution, axial ascending direction, without slice gap, TR = 11 ms, TE = 3 ms, flip angle = 8°).

#### 2.8.2 FMRI preprocessing and general linear model (GLM) analyses

FMRI data preprocessing and analysis was carried out with SPM12 (Wellcome Department of Cognitive Neurology, London, UK). Preprocessing followed the following steps: First, all functional images were simultaneously realigned to the first volume of the first run using septic b-spline interpolation and unwarped (using B0 maps) using the realign and unwarp function in SPM, followed by slice timing correction. T1-weighted structural images were then co-registered with the functional images and segmented into six different tissue classes using the segment function in SPM12. Next, all images were normalized to the Montreal Neurological Institute (MNI) T1 template with the forward deformation parameters obtained from segmentation. Lastly, all functional images were smoothed using spatial convolution with a Gaussian kernel of 6 mm at full width half maximum (FWHM).

Statistical analyses were carried out using the general linear model (GLM). Our statistical model reflects the factorial design of the current experiment by including separate regressors for each condition of the factor Threat (safe, threat) and Belief (false belief vs. outcome), separately for both the vignette and question periods. All regressors were modeled using a canonical hemodynamic response function (HRF). To best capture mentalizing during the question period, we used a variable epoch model from the onset of the question until option choice (button press). We also modeled regressors of no interest, which include each block cue, the feedback period, shock moments, and the Domain condition (economic games vs. life story). Note that we show in a previous paper that economic games and life story domains recruit similar social cognition regions during both periods (see Chang et al., 2023), which is why we include this factor here as a regressor of no interest. We also included omitted trials during which no response was made by the subject as a regressor of no interest. While omissions were rare (on average 0.55%), these trials were modeled explicitly to ensure that we only included trials on which we are certain participants paid attention to the task. Finally, the six motion parameters derived from the realignment procedure were modeled as regressors of no interest.

The main goal of the current study was to understand the impact of anxiety induced via threat of shock on the neural circuitry involved in mentalizing. The main focus of the analysis was the main effect reflecting the engagement of social cognition regions involved in mentalizing during (false) belief formation and inference, and the effect of Threat on these neural activations. We probed the mentalizing effect using the contrast Belief > Outcome. To address the effect of Threat on activity related to mentalizing, we performed two tests: given our previous results of suppressed mentalizing-related activity during trust decisions (Engelmann, Meyer et al., 2019), we expected similar suppressive effects in the current task that were assessed via the contrast Safe > Threat. However, threat can also enhance task-specific activation, as previously demonstrated via enhanced negative subjective value coding in the insula during risky decision making (Engelmann et al., 2015). We assessed this possibility by contrasting the Threat to the Safe condition (Threat > Safe).

Our study enables us to investigate these effects on two separate social cognitive processes, namely *belief formation*, which primarily occurs during the vignette period as subjects learn about the interactions between two partners, and *belief inferences*, which primarily occurs in the question period as subjects apply what they have learned to answer targeted questions about characters’ beliefs. To identify the effects of anxiety on activation patterns during belief formation and belief inferences, we first examined the main effect of the threat manipulation. To examine whether threat impacted the neural network involved in mentalizing, we conducted a conjunction analysis, which identified voxels that showed both, a mentalizing main effect (Belief > Outcome) and at the same time an effect of threat (Safe > Threat, Threat > Safe). We conducted the conjunction analyses separately for belief formation during the vignette period, and for belief-based inferences during the question period. Individual contrast maps that were entered into conjunction analyses were FWE-corrected at the cluster level with a cluster-forming threshold of *p* < 0.001. Conjunction analyses were conducted under the conjunction null hypothesis, requiring that all comparisons in the conjunction are individually significant (Nichols et al., 2005). Follow-up functional connectivity analyses were then conducted on those regions that are engaged during false belief formation and belief inferences and that show a simultaneous effect of Threat. To this end, we performed general psychophysiological interaction (gPPI) analyses to assess the extended circuitry that supports social cognition, and how this extended network in turn is impacted by Threat.

### 2.9 Connectivity analyses: generalized psychophysiological interaction (gPPI) analyses

To assess how threat impacts effective connectivity during mentalizing, we conducted generalized psychophysiological interaction (gPPI) analyses using the CONN toolbox (www.nitrc.org/projects/conn) (Whitfield-Gabrieli & Nieto-Castanon, 2012). To increase sensitivity and specificity for functional connectivity analyses, we again preprocessed the data in the CONN toolbox using the indirect segmentation and normalization pipeline in CONN, which is largely equivalent to our preprocessing steps above, but included the additional step of identifying and removing outlier scans from the analysis (ART, Whitfield Gabrieli). In accordance with the anatomical component-based noise correction method (Behzadi et al., 2007; Muschelli et al., 2014), additional denoising was conducted before functional connectivity analyses, which included 10 cerebrospinal fluid (CSF) and 10 white matter principal components as nuisance covariates, as well as motion parameters, their first temporal derivatives, and quadratic extensions (total 24 parameters, Friston et al., 1996), all scrubbed datapoints from the artefact removal step and all regressors and temporal derivatives. Low-frequency fluctuations were isolated using a low-pass temporal filter (0.008 Hz) after denoising. Generalized PPI analyses were thresholded using the Threshold-free cluster enhancement (TFCE) method (Smith & Nichols, 2009) using the CONN default setting (hmin = 1, E = 0.5, and H = 2) and peak-level family-wise error-corrected *p*-values.

Seed regions for functional connectivity analyses were extracted from two conjunction maps identifying overlap between the threat manipulation (threat > safe; safe > threat) and mentalizing (belief > outcome). These included (1) left medial temporoparietal junction (-42, -61, 26, k = 27) and the precuneus (-3, -55, 32, k = 45) during belief formation; and (2) bilateral TPJ (Left: -60, -61, 20, k = 83; Right: 60, -58, 35, k = 22), bilateral IFG (Left: -54, 23, 5, k = 29, Right: 51, 26, -7, k = 28), as well as left putamen (-28, 8, 5, k = 8) during belief inferences. Note that our method of determining seed regions for subsequent gPPI analyses based on activation clusters from conjunction analyses implies that ROIs have different sizes, but this method offers advantages in the current setting that includes both larger cortical and smaller subcortical regions that differ in their underlying anatomical size.

## 3 Results

### 3.1 Threat of shock manipulation check 1: skin conductance responses

We first tested the effectiveness of our threat of shock procedure to induce anxiety by assessing our participants’ autonomic arousal across the threat and safe blocks. Our results demonstrate a robust and larger SCR signal at the moment of receiving the shock (t_36_ = 10.67, *p* < 0.001) compared to baseline. We observed a similar effect when the block cue was presented, with significantly increased SCRs during threat relative to safety cues (t_36_ = 2.20, *p* = 0.03). Finally, increased SCRs were observed throughout the entire trial period, that is, from onset of the vignette until the end of the last trial, for threat relative to no-threat trials (t_36_ = 1.78, *p* = 0.002). Jointly, these results indicate that our threat of shock procedure induced increased autonomic arousal, both in terms of incidental anxiety that was triggered during the Shock Cue, and the prolonged anxiety of being under the threat of shock throughout Threat trials (Fig. 1B).

### 3.2 Threat of shock manipulation check 2: self-reported emotion

Next, we assessed what specific emotions are associated with the increased autonomic arousal observed during threat blocks using the emotion ratings provided by participants in the exit questionnaire. Since we were primarily interested in threat effects within each emotion category (and not across emotion categories), we restricted our analyses to the effects of Threat within each emotion category (e.g., anxiety threat vs. safe). To this end, we ran paired t-tests using a Bonferroni-corrected significance threshold (α = 0.0071). These revealed that self-reported emotions differed significantly between threat and safe blocks. Specifically, subjects reported higher aversive emotion during threat compared to safe blocks, including Anxiety (t_36_ = 7.57, *p* = 6.02 × 10^-9^, d = 1.24), Fear (t_36_ = 7.94, *p* = 2.00 × 10^-9^, d = 1.31), Disgust (t_36_ = 3.40, *p* = 1.65 × 10^-3^, d = 0.56), and Anger (t_36_ = 3.81, *p* = 5.18 × 10^-4^, d = 0.63). Moreover, during threat blocks, they felt more surprised (t_36_ = 5.56, *p* = 2.70 × 10^-6^, d = 0.91) and less happy (t_36_ = -6.45, *p* = 1.72 × 10^-7^, d = -1.06) compared to safe blocks. To further probe the effect of threat, we also tested whether threat of shock specifically increased self-reported anxiety to a larger extent than other aversive emotions using difference score (Δ shock-safe) for each emotion. We find that anxiety was reported to be more intense compared to all other aversive emotions, except fear and surprise: Anger, t_36_ = 4.53, *p* = 6.22 × 10^-5^, d = 0.75; Disgust, t_36_ = 5.04, *p* = 1.35 × 10^-5^, d = 0.83; Sadness, t_36_ = 6.60, *p* = 1.12 × 10^-7^, d = 1.08; Surprise, t_36_ = 2.15, *p* = 0.04, d = 0.35; Fear (t_36_ = 0.29, *p* = 0.78, d = 0.05). These results indicate that our paradigm successfully increased anxiety, and did so to a greater extent compared to other aversive emotions (anger, disgust, sadness) except for fear and surprise (Fig. 1B and C). Additionally, the effects of threat on self-reported emotion are not moderated by gender, as comparing gender effects within a given emotion and across threat categories does not yield any significant results (all p > 0.0051, Bonferroni-corrected alpha value for 14 comparisons = 0.00357).

In addition to significant psychophysiological and affective reactivity to shock administration, we also observe significant effects of electrical shock at the neural level. Specifically, administration of electrical shock led to significant activation within the pain matrix, including the insula, anterior cingulate cortex, and the right somatosensory cortex (see Fig. S2).

### 3.3 Behavioral results

**Fig. 2.**
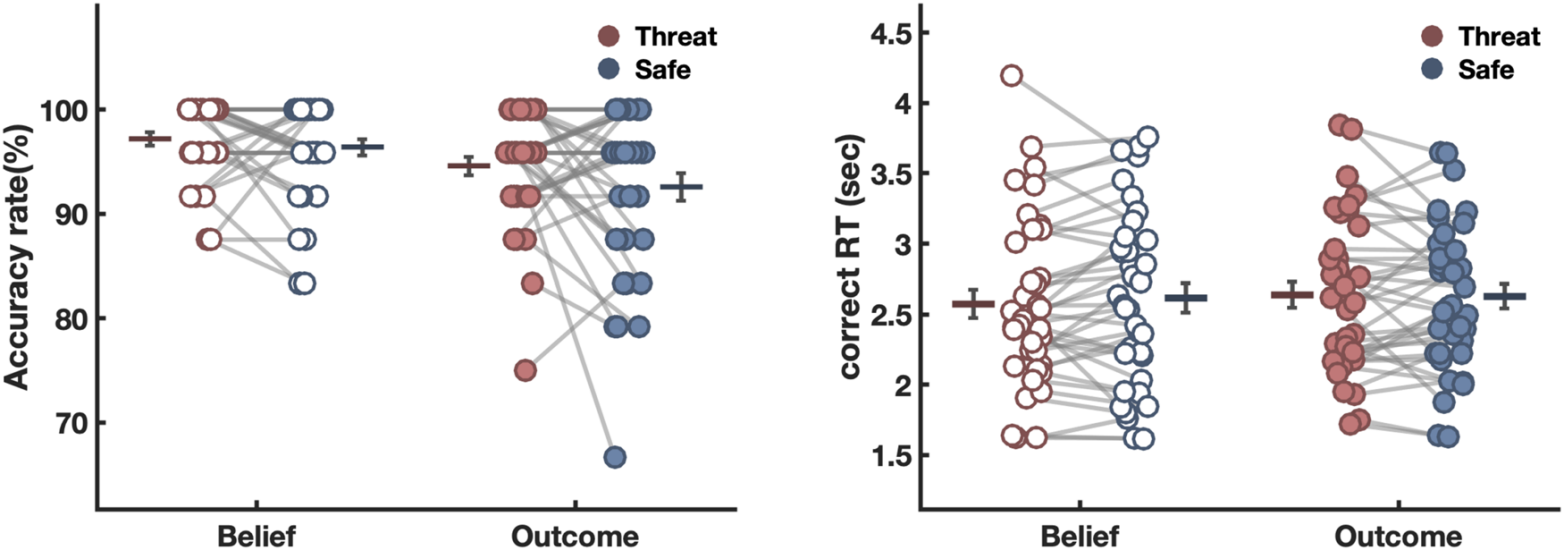
Mean of accuracy and mean RT in each condition. Average accuracy (A) and response time (B) for correct trials across Belief and Threat conditions reflecting averages calculated from the raw data. The horizontal line represents the mean, and error bars reflect the standard error of the mean (SEM) in each condition. Each dot represents an individual participant, and the gray connection lines between dots indicate that data are from the same subject.

Accuracy results are shown in Figure 2A. Results across different model specification show a significant main effect of Belief (*p* < 0.001), and no main effect of Threat (all *p* ≥ 0.124, across model specifications, see Table S1), or interactions with the factor Threat. The effect of Belief indicates better performance in the Belief condition (mean percent correct = 0.98) compared to the Outcome condition (mean percent correct = 0.96). Note that while significant, this effect is relatively small and reflects only a 2% improvement in the Belief compared to the Outcome condition.

Response time results, shown in Figure 2B, only showed a significant main effect for Belief (*p* = 0.035), but no main or interaction effects with the factor Threat (Table S1). The Belief main effect indicates that in the belief condition response times were slower compared to the outcome condition (mean log RT in belief = 0.882; mean log RT in outcome = 0.844). Together with the accuracy results, these findings suggest that participants in the belief condition are both better and slower, hinting at potential speed-accuracy trade-offs. To test for the possibility that speed-accuracy trade-offs distort our results, we computed the Balanced Integration Score that corrects for this possibility (BIS, Liesefeld & Janczyk, 2019) for each subject and in each condition. Results from this analysis are largely comparable with what we report in mixed models above (Table S2). Finally, we tested for gender effects: while gender does not moderate the accuracy results, response time results show that unlike females, males become faster under threat (Table S3). The behavioral results of individual participants in each condition are shown in Figure 2 below and reflect the mean performance of each participant across all trials within each condition.

### 3.4 FMRI results

#### 3.4.1 Mentalizing-related activity during belief formation and belief inferences

We observed a main effect of belief formation (contrast: belief>outcome during the vignette period) in the canonical social cognition network that includes bilateral temporoparietal junction (TPJ) (left: -48, -61, 26, k = 497; 48, right: -55, 23, -1, k = 380), dorsomedial PFC (-6, 47, 35, k = 422), right superior temporal gyrus (48, -28, -1, k = 554), and precuneus (-6, -55, 29, k = 125) (Fig. S3A) (Table 1).

**Table 1.** Activations for mentalizing effects during the vignette period.

Structure	L/R	Cluster size	x	y	z	Peak t
*Belief>Outcome (vignette)*						
Superior Temporal Gyrus	R	554	48	-28	-1	6.89
Temporal Parietal Junction (TPJ)	L	497	-48	-61	26	6.62
TPJ / Superior Temporal Gyrus	R	380	48	-55	23	6.39
Inferior Frontal Gyrus	L	122	-36	20	-19	6.26
Medial Prefrontal Cortex (mPFC)	L	422	-6	47	35	5.70
Middle temporal gyrus	L	292	-54	-4	-16	5.53
Precuneus	L	125	-6	-55	29	5.30
*Outcome>Belief (vignette)*						
Inferior parietal lobule	L	14022	-48	-37	41	-9.37
Inferior frontal gyrus	R	110	54	5	17	-5.91
Midbrain / thalamus	L	70	-9	-19	-4	-4.69

Upper panel: whole-brain analysis of the main effect of mentalizing during the vignette period at an FWE-corrected extent threshold of *p* < 0.05, initial cluster-forming height threshold *p* < 0.001 with a minimum cluster extend of 122 voxels. Lower panel: the reverse contrast for higher BOLD signal in the outcome compared to the belief condition at an FWE-corrected extent threshold of *p* < 0.05, initial cluster-forming height threshold *p* < 0.001 with a minimum cluster extent of 70 voxels.

When subjects made belief inferences (contrast: belief > outcome during the question period), we observed activity in left TPJ (extending to TP; -57, -28, -1, k = 3582), dmPFC (-9, 59, 32, k = 1343), precuneus (-3. -55, 29, k = 247), sensorimotor area (-48, -4, 50, k = 94), as well as cerebellum (24, -73, -37, k = 122; -27, -76, -40, k = 90) (Fig. S3B) (Table 2). Overall, activation patterns related to belief formation and belief inferences were very similar and included many of the same regions, but the latter had relatively more extensive activations.

**Table 2.** Activations for mentalizing effects during the question period.

Structure	L/R	Cluster Size	x	y	z	Peak t
*Belief > outcome (question)*						
Superior temporal gyrus/lTPJ	L	3582	-57	-28	-1	12.68
Lateral temporal cortex	R	2857	54	5	-25	10.41
Medial prefrontal cortex	L	1343	-9	59	32	8.39
Cerebellum	R	122	24	-73	-37	6.36
Sensorimotor cortex	L	94	-48	-4	50	6.27
Precuneus	L	247	-3	-55	29	6.27
Cerebellum	L	90	-27	-76	-40	6.01
Primary motor cortex	R	158	45	-19	59	4.59
Superior frontal gyrus	L	165	-27	11	56	7.95
*Outcome > belief*						
Supramarginal gyrus/intraparietal sulcus	R	593	54	-40	50	7.88
Inferior parietal lobule	L	149	-42	-43	41	6.76
Precuneus	L	398	-27	-70	41	6.66
Middle frontal gyrus	R	137	27	11	59	6.41
Middle and inferior temporal lobe	R	146	54	-46	-13	6.19

Upper panel: whole-brain analysis of the main effect of mentalizing during the question period at an FWE-corrected extent threshold of *p* < 0.05, initial cluster-forming height threshold *p* < 0.001 with a minimum cluster extent of 90 voxels. The lower panel shows the reverse contrast reflecting higher BOLD signal in the outcome compared to the belief condition at an FWE-corrected extent threshold of *p* < 0.05, initial cluster-forming height threshold *p* < 0.001 with a minimum cluster extent of 137 voxels.

#### 3.4.2 Effects of threat on activation patterns during belief formation and belief inferences

During belief formation, we identified increased threat-related activity (contrast: threat > safe during the vignette period) in bilateral intraparietal sulcus (-27, -61, 35, k = 6522), which includes subclusters in ACC (18, 26, 35), dorsolateral PFC (-33, 14, 56), and cuneus / precuneus (27, -67, 35) (Fig. S4A). Many of these regions overlap with canonical attention regions, as identified via a conjunction analysis between the current results and the neurosynth meta-analysis for attentional control (see Fig. S5A). No significant suppression was found during belief formation in the vignette period (contrast: safe > threat during the vignette period, Table 3).

**Table 3.** Activations reflecting threat effects during the vignette period.

Structure	L/R	Cluster size	x	y	z	Peak t
*Main effect: Threat > Safe*						
Intraparietal sulcus	L	6522	-27	-61	35	7.27
*Corpus callosum (subcluster)*	*R*		*24*	*23*	*20*	*7.04*
*Anterior cingulate cortex (subcluster)*	*R*		*18*	*26*	*35*	*6.40*
*Fusiform gyrus (subcluster)*	*R*		*24*	*-64*	*-10*	*5.99*
*V1 (primary visual cortex) (subcluster)*	*R*		*-27*	*-79*	*11*	*5.99*
*Parahippocampal gyrus (subcluster)*	*R*		*33*	*-34*	*23*	*5.97*
*DLPFC (subcluster)*	*L*		*-33*	*14*	*56*	*5.92*
*Parietal lobule (subcluster)*	*L*		*-24*	*-76*	*41*	*5.89*
*Fusiform gyrus (subcluster)*	*L*		*-38*	*-67*	*-10*	*5.81*
*Middle occipital lobule*	*L*		*-33*	*-78*	*0*	*5.73*
*Premotor cortex / frontal eye field*	*L*		*-42*	*-7*	*53*	*5.67*
*Angular gyrus*	*R*		*42*	*-64*	*35*	*5.65*
*Corpus callosum (subcluster)*	*L*		*-21*	*28*	*23*	*5.63*
* Cuneus / precuneus (subcluster)*	*R*		*27*	*-67*	*35*	*5.56*
* Intraparietal sulcus (subcluster)*	*R*		*33*	*-70*	*35*	*5.47*
R. Middle / Superior frontal gyrus	R	138	36	5	53	5.33
*Main effect: Safe > Threat*						
No significant activations						

Upper panel: whole-brain analysis of the main effect of threat enhancement during the vignette period at an FWE-corrected extent threshold of *p* < 0.05, initial cluster-forming height threshold *p* < 0.001 with a minimum cluster extend of 138 voxels. Lower panel: no significant effect was found for threat-related suppression.

During belief inferences, we identified significant suppression due to threat (contrast: safe > threat during the question period) in bilateral TPJ (-63, -52, 38, k = 151; 54, -46, 53, k = 139), bilateral inferior frontal gyrus (-42, 20, 5, k = 79; 54, 29, -7, k = 64), and left putamen (-15, -13, 2, k = 330) (Fig. S4B). No enhanced activity was found during belief inferences in the question period (contrast: threat > safe during the question period Table 4).

**Table 4.** Activations reflecting threat effects during the question period.

Structure	L/R	Cluster size	x	y	z	Peak t
*Main effect: Safe > Threat*						
L. supramarginal gyrus / TPJ	L	151	-63	-52	38	5.67
L. inferior frontal gyrus	L	79	-42	20	5	5.07
L. thalamus / putamen	L	330	-15	-13	2	4.93
R. supramarginal gyrus / TPJ	R	139	54	-46	53	4.87
R. inferior frontal gyrus	R	64	54	29	-7	4.60
*Main effect: Threat > Safe*						
No significant activations						

Upper panel: whole-brain analysis of the main effect of threat suppression during the question period at an FWE-corrected extent threshold of *p* < 0.05, initial cluster-forming height threshold *p* < 0.001 with a minimum cluster extent of 64 voxels. Lower panel: no significant effect was found for threat-related enhancement.

#### 3.4.3 Effects of anxiety on activations reflecting belief formation and belief inferences

To assess to what degree the effects of threat on activations during belief formation and belief inferences affected specifically social cognition regions involved in mentalizing, we first probed our fMRI data for the interaction between the factors Threat and Belief. We did not find significant interaction effects during either belief formation or belief inferences, reflecting that anxiety did not *specifically* impact activity related to mentalizing. To probe our data for non-specific effects of anxiety, we performed conjunction analyses that assessed whether the threat-related enhancements during belief formation and suppressions during belief inferences occur in mentalizing regions. To this end, we multiplied the FWE-corrected maps (cluster-forming threshold of *p* < 0.001 with FWE cluster-level correction) for the main effects of mentalizing (belief > outcome) and threat enhancement (threat > safe), as well as threat suppression (safe > threat) from the previous analyses.

**Fig. 3.**
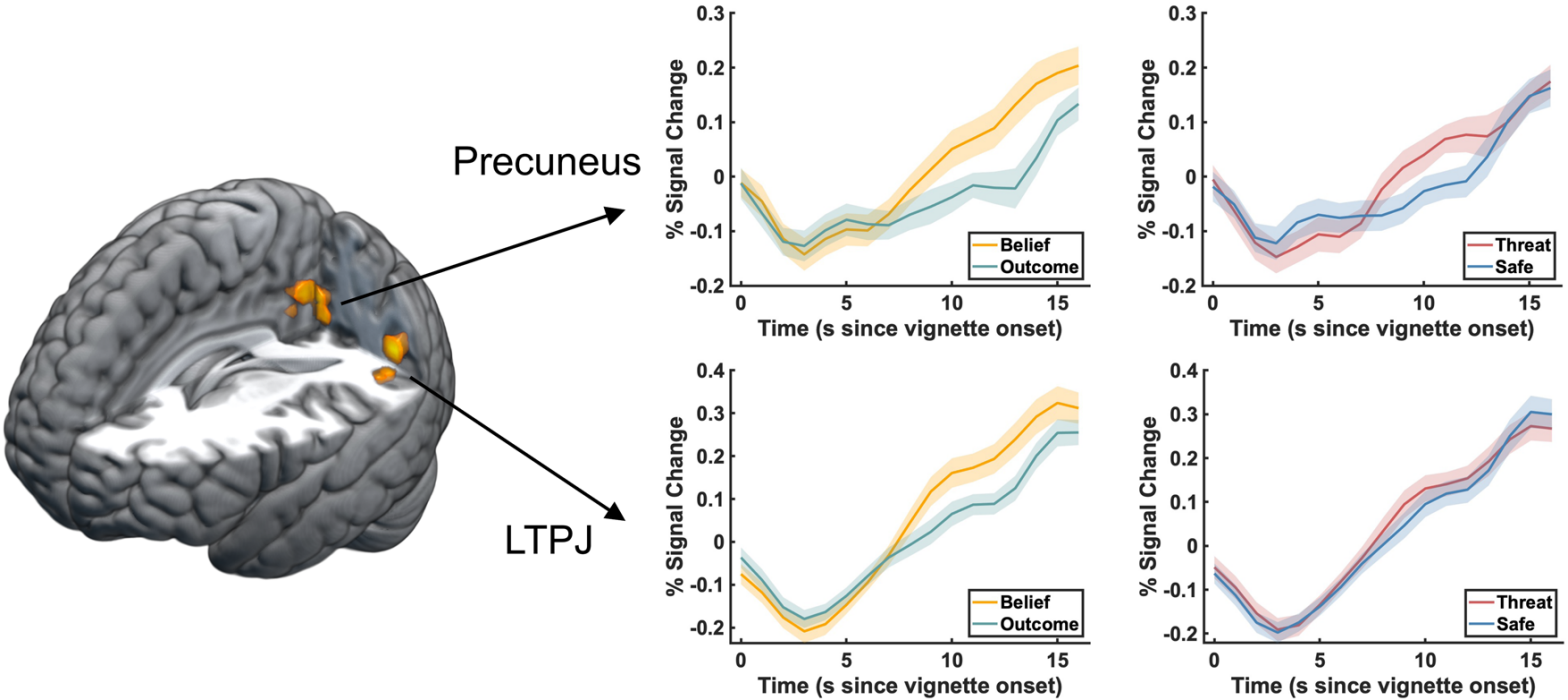
Conjunction analysis of the activations reflecting belief formation and threat-enhancement during the vignette period. Areas are shown that activate during mentalizing (belief > outcome) and, at the same time, show enhancement by threat (threat > safe) during belief formation. Two regions were identified in this conjunction analysis, including medial left TPJ and precuneus (also see Table 5). Inlets show time courses of significant activations plotted separately for the mentalizing and threat main effects. Time courses were extracted from all voxels in the regions identified by conjunction analyses.

During belief formation, we found significant overlap between regions that showed *enhanced* activity due to threat and regions involved in mentalizing. These regions include the left medial temporoparietal junction (-42, -61, 26, k = 27) and the precuneus (-3, -55, 32, k = 45). Figure 3 (and Table 5) shows these regions together with the time courses for the two main effects that simultaneously show enhanced belief-based activation as well as enhanced threat related activation. We did not find any significant results reflecting conjoined activity for mentalizing and threat *suppression* during belief formation (Table 5).

**Table 5.** Conjunction analysis of mentalizing and threat effects during the vignette and question period respectively.

Structure	L/R	Cluster Size	x	y	z
*Conjunction of mentalizing effect and the enhancement by threat during the vignette period*					
Superior Temporal Gyrus	L	27	-42	-61	26
Precuneus	L/R	45	-3	-55	32
*Conjunction of mentalizing effect and the suppression by threat during the vignette period*					
*No significant activations*					
*Conjunction of mentalizing effect and the enhancement by threat during the question period*					
*No significant activations*					
*Conjunction of mentalizing effect and the suppression by threat during the question period*					
Inferior frontal gyrus	L	29	-54	23	5
Putamen	L	8	-28	8	5
Temporoparietalparietal junction	L	83	-60	-61	20
Inferior frontal gyrus	R	28	51	26	-7
Temporoparietalparietal junction	R	22	60	-58	35

Results from conjunction analyses identifying regions simultaneously showing (1) enhanced mentalizing-related and enhanced threat-related activity during the vignette period, and (2) enhanced mentalizing-related and suppressed threat-related activity in the question period. Individual maps that were entered into conjunction analyses were thresholded at cluster level with an FWE-corrected alpha value <= 0.05. Conjunction analyses were conducted under the conjunction null hypothesis, requiring that all comparisons in the conjunction are individually significant (see Nichols et al., 2005).

**Fig. 4.**
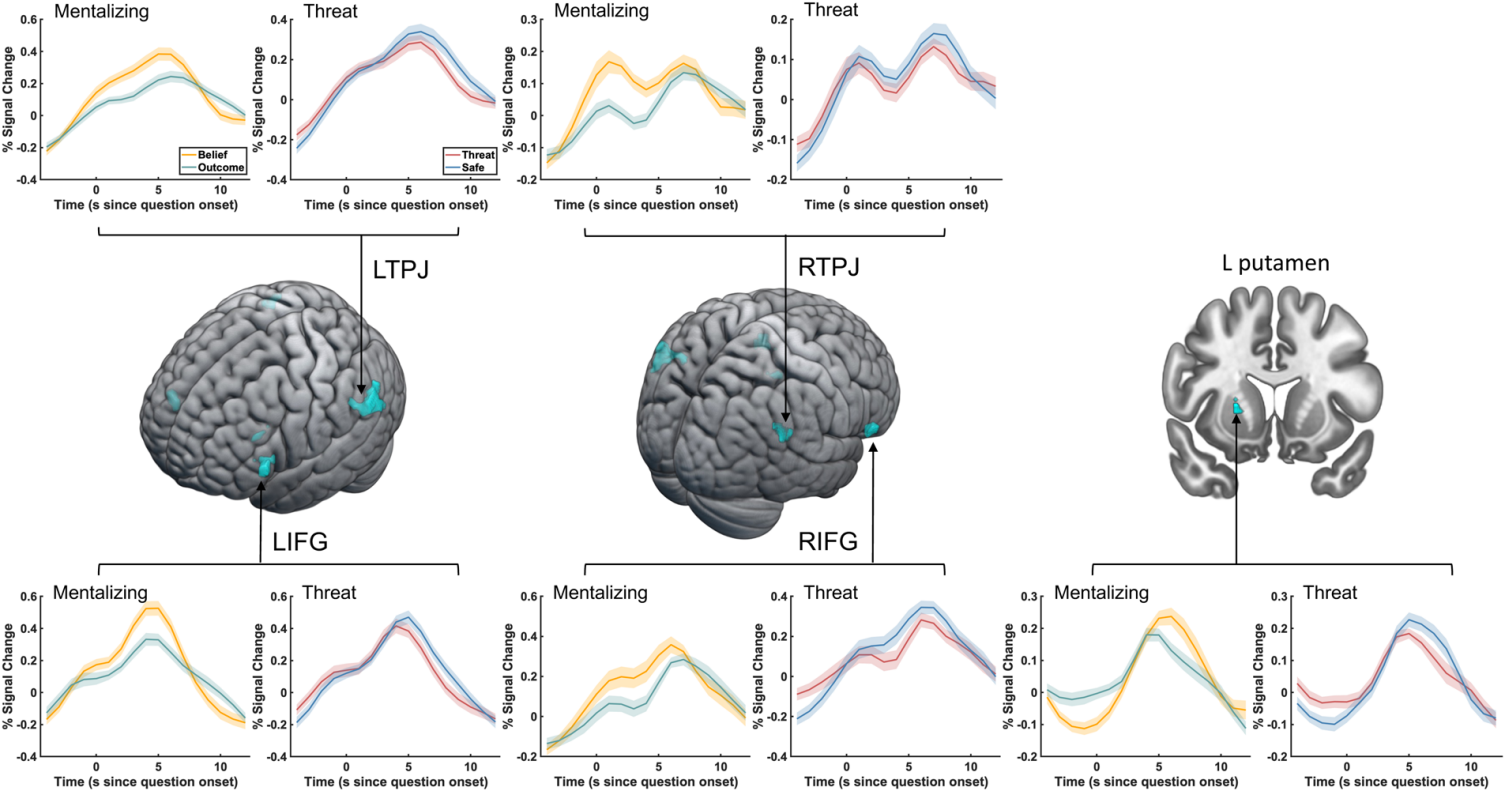
Conjunction analysis of the activations reflecting belief inferences and threat-suppression during the question period. Areas are shown that activate during mentalizing (belief > outcome) and, at the same time, show suppression by threat (safe > threat) during belief inferences. Five main clusters were identified in this conjunction analysis, including bilateral IFG, bilateral TPJ, and left putamen (also see Table 5). Inlets show time courses of significant activations plotted separately for the mentalizing and threat main effects. Time courses were extracted from all voxels in the regions as identified by conjunction analyses.

During belief inferences, we found significant overlap between regions that are significantly *suppressed* by threat and regions that are involved in mentalizing. The conjunction map is shown in Figure 4 (also see Table 5) and reveals overlap in bilateral TPJ (left: -60, -61, 20, k = 83; right: 60, -58, 35, k = 22), bilateral IFG (left: -54, 23, 5, k = 29; right: 51, 26, -7, k = 28), and also left putamen (-28, 8, 5, k = 8). Time courses were extracted from clusters that show significant conjunction and are plotted separately for the mentalizing and threat main effects. We did not find any significant results reflecting conjoined activity for mentalizing and threat *enhancement* during belief inferences (Table 5).

### 3.5 Functional connectivity results using general psychophysiological interaction (gPPI) analyses

**Fig. 5.**
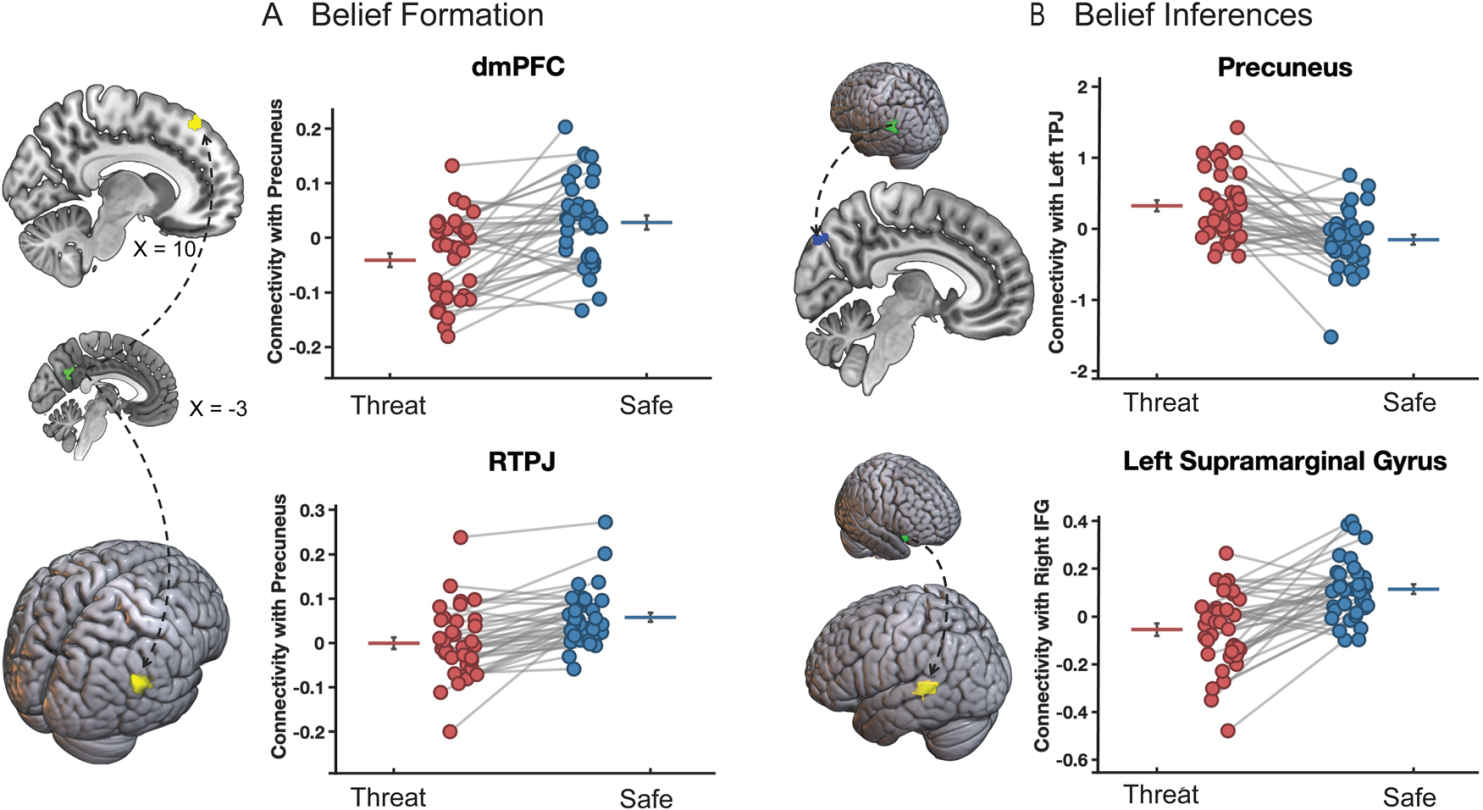
Main effect of threat on connectivity during belief formation (A) and belief inferences (B). (A) During belief formation in the vignette period, the connectivity between the precuneus seed region and dmPFC and rTPJ target regions was significantly suppressed by Threat. (B) During belief inferences in the question period, the connectivity between left TPJ and precuneus was enhanced by Threat, while a threat-related suppression was found between right IFG and left SMG.

To identify the effects of anxiety on connectivity between regions showing activity that reflects both mentalizing and threat-related changes in activity, we conducted generalized PPI analyses using as seeds the regions from the conjunction analyses reported in Figure 3 for belief formation and in Figure 4 for belief inferences. We first tested the main effect of threat on connectivity during belief formation and belief inferences. During belief formation, the main effects of threat identified suppressed connectivity within the social cognition network (safe > threat during the vignette period). As shown in Figure 5A, we found threat-induced suppression in connectivity between the precuneus seed and its targets in right TPJ (58, -52, 26, k = 243), and a posterior region of dmPFC (16, 30, 50, k = 138). We did not find any *increased* connectivity as a function of threat during belief formation. During belief inferences, shown in Figure 5B, threat suppressed the connectivity between the right IFG seed and its target in the left supramarginal gyrus (-56, -42, 8, k = 247), and increased connectivity between the left TPJ seed and its target in posterior precuneus (6, -82, 38, k = 136).

### 3.6 Specific suppression of belief-based connectivity

**Fig. 6.**
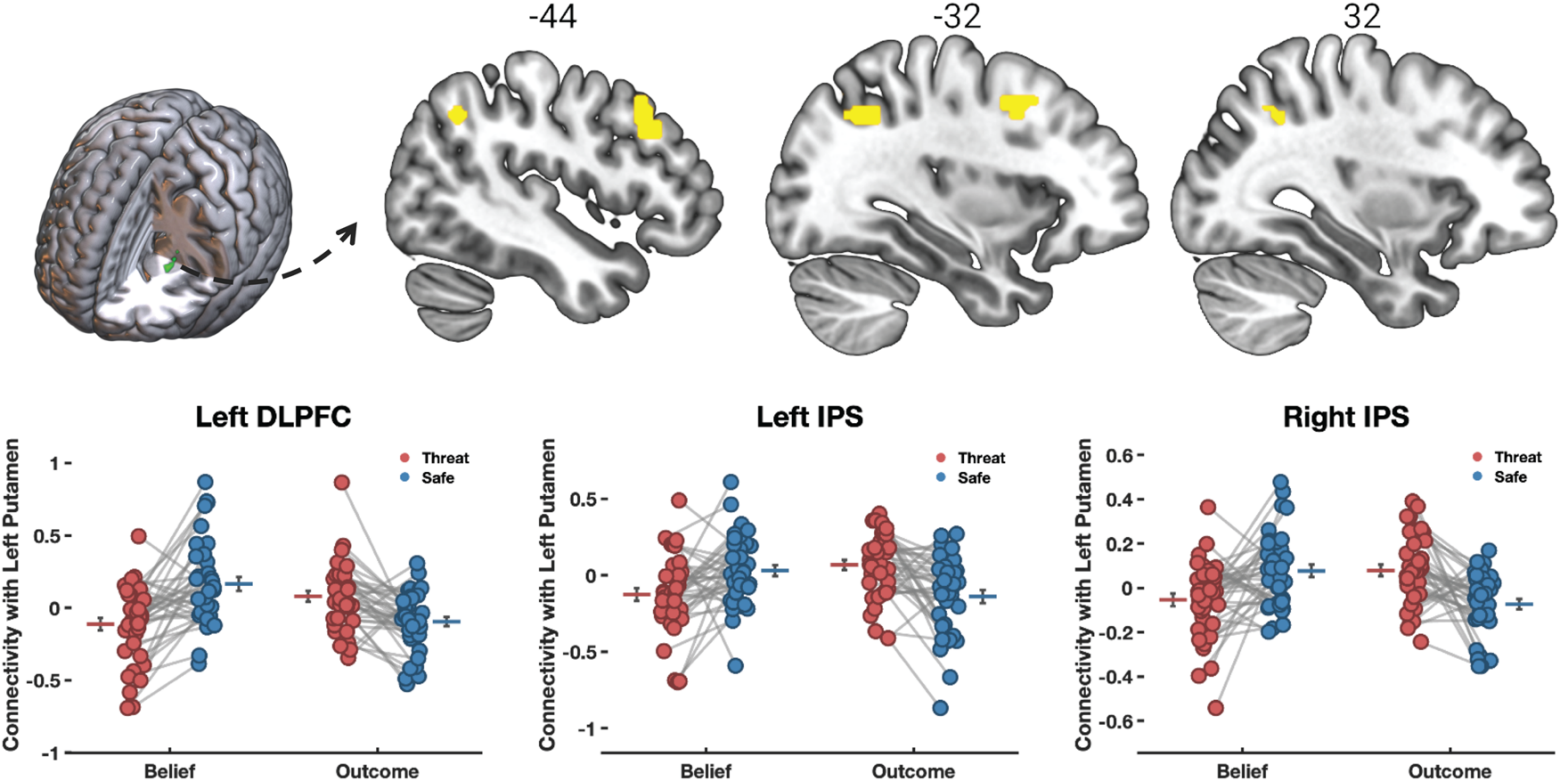
Threat specifically suppressed connectivity between left putamen and its targets in IPS and dlPFC. The figure shows significant threat-related suppression of belief-based connectivity during belief inferences in the question period, reflected by the interaction between the Threat and Belief factors. Connectivity between the putamen seed region and its targets in IPS and dlPFC is specifically suppressed in the Belief condition, and shows the opposite trend in the Outcome condition within this network.

After identifying the main effect of threat on connectivity, we next conducted follow-up analyses probing for specific effects of Threat on belief-based connectivity compared to outcome-based connectivity via an interaction analysis. During belief formation, we were not able to identify such specific effects. However, during belief inferences, we found significant threat-related suppression of connectivity in the belief but not the outcome condition between the left putamen seed and its targets in bilateral intraparietal sulcus (IPS; right: 24, -52, 38, k = 139; left: -32, -60, 38, k = 168) and left dlPFC (-42, 18, 44, k = 334, Fig. 6). These regions fall within the canonical attention network (see conjunction analysis in Fig. S5B), which tentatively suggests reduced connectivity with attentional control regions when participants made inferences about others’ beliefs under conditions of anxiety.

**Fig. 7.**
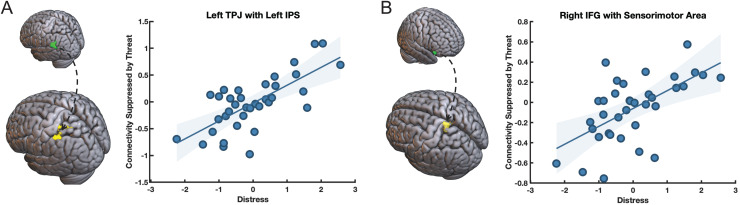
Suppression of connectivity between left TPJ and left IPS and right IFG and left sensorimotor cortex during belief inferences is moderated by dispositional distress. (A) the left TPJ seed region (green) shows an increase in threat-related suppression of connectivity (threat < safe) with its target in left IPS (yellow) as a function of dispositional distress. (B) The right IFG seed region (green) shows an increase in threat-related suppression of connectivity with its target in the left sensorimotor cortex (yellow) as a function of dispositional distress. The y-axis reports the main effect of threat (safe - threat), such that reduced connectivity under threat compared to safety leads to greater contrast values reflecting enhanced threat-related suppression. Distress is a latent variable obtained from factor analyses (see Table S4).

Exploratory connectivity-behavior correlation analyses further confirm the suppressed effective connectivity between mentalizing and attentional control regions (Fig. 7). Specifically, we inspected the relationship between threat-related connectivity (threat main effect contrast: safe > threat) and dispositional distress. Dispositional distress is a latent variable obtained from factor analyses of multiple questionnaires measured before the start of the experiment (for a detailed description, see the factor analysis section in the Supplementary Materials). Distress has particularly high loadings (>0.7) on the State-Trait Anxiety Inventory (STAI), NEO5 neuroticism, Beck’s Depression Inventory (BDI), and the Perceived Stress Scale (PSS) (see the factor analysis results reported in the supplement in Table S4 and Fig. S6). We found that dispositional distress significantly modulates threat-related suppression of connectivity between the left TPJ seed and left IPS, as well as between the right IFG seed and left sensorimotor cortex. These results indicate that left TPJ – IPS (and IFG-PCG) connectivity is suppressed to a larger extent for subjects reporting significantly larger levels of trait distress.

## 4 Discussion

The current experiment investigated the impact of anxiety on the neural signature of mentalizing by asking participants to complete two versions of the false-belief task, an economic games and a standard version, under the threat of electrical shock. We observed a clear effect of threat on arousal and self-reported emotion, but found little effect of threat on behavior. Our fMRI results both replicate and extend prior work on the neural signature of mentalizing. First, we replicate prior results showing that mentalizing is associated with activity in areas within the social cognition network that includes bilateral temporal pole, right superior temporal gyrus, bilateral TPJ, precuneus / PCC, and also mPFC (see Fig. S3, and our previous analysis of this data reported in Chang et al., 2023). Similar activation patterns related to mentalizing occur during both phases of the current task, namely during belief formation in the vignette period, and belief inferences in the question period. Importantly, we extend prior work by demonstrating the effects of anxiety on the neural correlates of belief formation and belief inferences. When contrasting threat of shock to safety, our results demonstrate (1) enhanced activity in a medial region of TPJ and the precuneus under threat during belief formation, and (2) suppression of activity in a wider social cognition network during belief inferences. The latter finding suggests that regions that are involved in taking the perspective of others are the same regions that show suppression under conditions of threat and anxiety. We tested this via a conjunction analysis between the contrast Belief vs. Outcome, reflecting mentalizing, and the contrast Safe vs. Threat, reflecting threat suppression. We found significant overlap between mentalizing and threat suppression in bilateral TPJ, bilateral IFG, and left putamen during belief inferences (Fig. 4). This finding is consistent with our hypothesis that anxiety suppresses the neural underpinnings of mentalizing. However, our results indicate that the suppressive effects of anxiety are non-specific given the absence of significant interactions between Threat and Belief in our behavioral and fMRI models. In line with our previous fMRI study investigating the impact of anxiety on the neural correlates of trust decisions (Engelmann, Meyer et al., 2019), we show here that anxiety also suppressed TPJ and IFG activity when participants made inferences about others’ beliefs in two versions of the false belief task. These results therefore lend further support to the notion that one effect of anxiety is to suppress activity in social cognition regions when current task demands draw on mentalizing abilities. Taken together, we confirm that across different tasks that include economic games, false belief tasks, and the RMET, all of which rely on mentalizing abilities, one consistent effect of anxiety across studies is to suppress the activity and connectivity of social cognition regions (see also Engelmann, Meyer et al., 2019; Nolte et al., 2013).

The finding that bilateral TPJ is suppressed in the presence of threat confirms and extends previous results demonstrating suppressed TPJ activity after social stress induction during RMET performance (Nolte et al., 2013), and suppressed activity and connectivity during trust decisions under threat of shock (Engelmann, Meyer et al., 2019). The TPJ has been repeatedly implicated in mentalizing (Decety & Lamm, 2007; Mar, 2011; Mitchell, 2009; Molenberghs et al., 2016; Schurz et al., 2014; Van Overwalle, 2009) and in the current study we replicate this result in the context of a standard false belief task and a recent extension of this task that describes bilateral interactions in economic games (Chang et al., 2023). Importantly, we find that the TPJ simultaneously shows a main effect of mentalizing and of threat, particularly during belief inferences. Moreover, the activation clusters of these main effects overlap spatially as identified via conjunction analyses. Jointly, these results suggest threat-related, but non-specific suppression of activity in the TPJ during belief inferences.

The finding of suppressed activity in IFG under conditions of threat is consistent with its role in inhibitory control (Aron et al., 2014; Bari & Robbins, 2013; Hampshire et al., 2010). This region is also part of an extended social cognition network (e.g., Arioli et al., 2021) and likely plays a role in mentalizing by inhibiting self-referential thought to enable taking the perspectives of others (Lieberman, 2007). This antagonistic connection between self-referential thought and mentalizing has been confirmed by a number of prior fMRI (Van der Meer et al., 2011), and lesion studies (Samson et al., 2005). Moreover, IFG activity during mentalizing has been shown to be suppressed in the context of anxiety and stress, including in participants with anxious attachment style (Schneider-Hassloff et al., 2015), and those undergoing attachment-related stress induction (Nolte et al., 2013). In the same vein, our conjunction analysis showing simultaneous main effects of mentalizing and threat suggests that anxiety can disrupt mentalizing processes by suppressing inhibitory regions such as IFG.

Finally, we found differential effects of threat during belief formation and belief inferences. Specifically, during the vignette period, when participants read vignettes and formed beliefs about the agents, we found *enhanced* activity in task-related network commonly associated with attention. During the question period on the other hand, when participants made belief-based inferences about the agents in the vignette, we identified the hypothesized *suppression* of activity in social cognition regions. Jointly, these results indicate that incidental anxiety has opposing effects on brain activity in different networks during the two task phases. We speculate that the enhanced activity observed under threat during belief formation in the dorsal attention network is a non-specific effect that indicates enhanced sustained attention to the environment due to the possibility of receiving an electric shock throughout this prolonged period (Engelmann et al., 2015). An alternative explanation for these effects of incidental anxiety would be an enhanced attention to the task at hand (vignette reading), which is not reflected by current behavioral results and therefore not a likely explanation.

The threat-related increase in activation in social cognition regions during belief formation that was identified by the conjunction analysis reported in Figure 3 parallels results from prior meta-analyses showing hyperactivation in similar regions of social anxiety disorder patients (Bruehl et al., 2014; Yoon et al., 2016). Such hyperactivation within the mentalizing network has been observed during self-referential processing in patients with SAD (Yoon et al., 2016). Extrapolating from these results to the current findings suggests that the increased activation in TPJ and PCC under threat may reflect an enhanced effort to focus on and understand the beliefs of others to compensate for an increase in egocentric perspective-taking under conditions of heightened threat (e.g., Silani et al., 2013; Soutschek et al., 2016; Todd et al., 2015). While intriguing, this interpretation is highly speculative given the current data, but may provide an interesting avenue for future research.

On the other hand, the result of threat-related suppression of activity in social cognition regions when participants made belief-based inferences in the question period supports our prior findings (Engelmann, Meyer et al., 2019). This result is also consistent with our hypothesis that one effect of threat is to suppress specifically those regions that are involved in solving ongoing task demands. In the current task, these demands are fulfilled by regions involved in taking the perspective of others. Jointly, the current fMRI results thus tentatively support the notion that threat causes non-specific attentional effects to a threatening environment during belief formation accompanied by egocentric perspective-taking, and subsequently suppresses activity in social cognition regions during belief inferences. While these results are consistent with prior reports (Engelmann, Meyer et al., 2019), alternative explanations are possible that need to be further evaluated in future research.

### 4.1 Connectivity results

Our functional connectivity analyses showed largely suppressive effects of anxiety. We found threat-based suppression of connectivity both during belief formation, specifically between the precuneus seed region and target regions in dmPFC and rTPJ, as we all as during belief inferences, where the connectivity between right IFG and left SMG was suppressed. These main effects reflect that connectivity is largely suppressed by threat during belief formation and subsequent inferences. One exception is the connectivity between left TPJ and posterior precuneus, which was enhanced by threat during belief inferences. Such non-specific effects of threat could indicate an enhanced effort in sustaining attention to the task at hand in the presence of threat, but also enhanced attention to a threatening environment due to the possibility of receiving an unpredictable electric shock throughout a prolonged period. The connectivity patterns between different parts of the precuneus with various nodes of the social cognition network in our study aligns with the notion that the precuneus may be functionally heterogenous (Willbrand et al., 2022). This resonates with previous research that reported a dissociation between the ventral and dorsal parts of the PCC (Leech et al., 2011), demonstrating suppressed connectivity between the ventral-PCC and nodes of the default mode network (DMN) under high-cognitive load, whereas dorsal-PCC showed increased connectivity with the DMN in the same condition.

In addition to these non-specific effects of threat, we also found specific threat-related suppression during belief inferences, that is, connectivity that is related to mentalizing, and specifically suppressed in the belief relative to the outcome condition. Such interaction effects were identified for the connectivity between the left putamen seed and its targets in IPS and dlPFC. They reflect threat-based suppression of connectivity during mentalizing, but not during reasoning about events that happen to another person and economic game payouts during the outcome condition. Our functional connectivity results therefore demonstrate an interaction between the reward system, represented by the putamen seed, and frontoparietal attentional systems, represented by IPS and dlPFC, when participants engage in social cognition under threat. In a conjunction analysis reported in Figure S5, we show that these target regions overlap with attentional control regions as identified by a neurosynth meta-analysis. These results point to the possibility that social cognitive processes receive support from additional networks, particularly attentional control regions, and that these connections are suppressed during threat. This notion is further supported by behavior-connectivity analyses, which showed that connectivity between left TPJ and left IPS is moderated by dispositional distress. Specifically, during threat, connectivity is suppressed to a larger extent for subjects showing higher levels of dispositional distress, which is closely related to trait anxiety. Jointly, these results indicate an interesting interaction between mentalizing and attentional regions that is moderated by the presence of threat and the dispositional distress of a person.

One interpretation of the current results of reduced interconnectivity between social cognition and attentional control regions rests on results commonly reported for anxiety disorder, namely threat-related attentional biases, such as difficulties in disengaging attention from threatening and invalid cues (e.g., Pacheco-Unguetti et al., 2011; Yiend & Mathews, 2001). Meta-analyses show that such attentional biases are reliably found in anxious individuals (Bar-Haim et al., 2007; Cisler & Koster, 2010). Given that attentional networks are disrupted in anxiety disorders (Sylvester et al., 2012; Williams, 2016), and that such disruption is associated with the extent of dispositional anxiety (Forster et al., 2015), the reduced connectivity between attention and social cognition networks could reflect a reduced flexibility in social cognitive processes under conditions of anxiety. These effects may be driven by enhanced attention to the threatening context, which limits the cognitive resources that can be dedicated to solving ongoing task demands (see also Engelmann et al., 2015; Engelmann, Meyer et al., 2019).

Moreover, the current study enables tests of interactions between anxious predispositions and anxiety-evoking situations. Our results showing enhanced suppression of connectivity under conditions of threat in individuals with high levels of dispositional distress (which has particularly high loadings on trait anxiety and neuroticism, see Table S4) suggest that trait anxiety can moderate the effects of stressful situations on the interactions between social cognition and attention networks that address ongoing behavioral demands. Previous results from meta-analyses showing consistent but overall small reductions in mentalization capacities in anxiety disorder (Chevalier et al., 2023; Sloover et al., 2022 suggest that such mentalization capacity limitations may surface mostly in stressful and anxiety-evoking contexts. Our behavior-connectivity analyses, showing reductions in the functional connectivity between networks that support ongoing task demands, lend indirect support to the notion that stressful and anxiety-evoking situations can reduce mentalizing abilities, particularly in anxious individuals.

However, our discussion of the connectivity results is rather speculative at this point and future research is needed to identify the specific interactions between social cognitive and attentional networks. Important tasks for future research will be to identify (1) how stressful and threatening contexts differentially impact behavior and task-relevant neural networks in individuals with varying levels of trait anxiety, and (2) how activation and connectivity patterns relate to behavioral changes, which we were not able to identify in the current study due to overall high-performance levels.

### 4.2 Limitations

Despite careful experimental design and well-matched control conditions, a number of potential limitations are worth mentioning. First, while we calibrated the task in two pilot studies (reported in Chang et al., 2023), performance in the scanning environment improved and was close to ceiling level. The false belief task used here was therefore not optimal for revealing the behavioral effects of threat. Future studies on the effects of threat on mentalizing performance should therefore consider a more difficult task to maximize their ability to detect the behavioral effects of threat. This could be accomplished by redesigning the questions from the current task (for instance by including additional answer options), or using a more difficult task such as reasoning about others in a betting game (Carpenter & Robbett, 2022). On the flip side, however, current results are consistent with previous observations that experimental treatments, while having similar effects on behavior, can differentially impact mental operations and the activation patterns associated with those (Engelmann, Meyer et al., 2019; Hein, Morishima et al., 2016; Wilkinson & Halligan, 2004). Moreover, since the purpose of the current study was to understand the effects of threat in terms of brain activity and connectivity, it can be desirable that there are no differences in behavioral responses across conditions as this reduces performance-based distortions of the fMRI signal (Engelmann, Meyer et al., 2019; Hein, Morishima et al., 2016; Wilkinson & Halligan, 2004). Furthermore, it should be mentioned that the outcome condition in the economic-games context is associated with slightly reduced accuracy. This condition, in which participants were asked to compute the payoffs in trust and ultimatum game interactions, served as an important control condition for the economic-games false belief task. Our approach in the current paper was to collapse across the two version of the false-belief task we included here to enhance statistical power, as we show in a previous paper that these two tasks lead to very similar activation patterns within the social cognition network (for detailed discussion, see Chang et al., 2023). Moreover, we control for this condition in our analyses via the inclusion of the Task Domain regressor in all behavioral and fMRI models.

A further limitation of our study is the lack of jitter between the vignette and question periods of the task. Jitter would have allowed for better separation of the hemodynamic response across these periods. Despite this, our decision not to include jitter was based on several factors: (i) to facilitate comparison with previous studies (e.g., Saxe & Kanwisher, 2003; Young et al., 2010), (ii) to reduce the cognitive burden on participants, and (iii) to keep the experiment duration reasonable. Furthermore, this limitation is mitigated by the BOLD patterns observed in Figures 4 and 5, which show the expected responses during both task periods. Specifically, during the vignette period, BOLD responses peak at about 15 s, reflecting sustained social cognitive processes, while during the question period, they peak at around 5 s, reflecting transient social cognitive processes consistent with the average response time during that period. We show similar time courses in our previous analyses of the social-cognitive aspects of this dataset (see Chang et al., 2023), further mitigating this concern.

## 5 Conclusion

Anxiety is a ubiquitous emotion that can have important adaptative functions. Recent research has demonstrated that anxiety can have wide-ranging effects on cognitive processes that support ongoing behavior, including attention, memory, and learning. This makes much sense, considering that one role of anxiety is to increase vigilance and attention to avoid potentially adverse outcomes. Despite the extensive prior research on the impact of anxiety on cognition, we identified a gap in the neurobiological literature on the effects of anxiety on social cognitive processes. We address this gap in the current experiment by inducing anxiety using the well-established threat-of-shock procedure and assessing its behavioral and neural impact on mentalizing. Mentalizing is an important social cognitive process that enables us to understand the beliefs and intentions of others and that is consistently associated with activity in a core social cognition network consisting of bilateral TPJ, dmPFC, and precuneus. Our results suggest that incidental anxiety suppressed the activity and connectivity within the social cognition network during mentalizing, but did so in a non-specific manner. Our work further provides novel evidence for the involvement of the frontal-parietal attentional network during mentalizing, which showed threat-related suppression of connectivity during mentalizing, but not during the control condition. These results underline the importance of large-scale network interactions between social cognitive and attentional networks, further indicating that social cognitive processes are supported by attentional processes. One intriguing suggestion from our results is that the reward system in general, and the putamen in this particular case, may serve as a hub enabling cross-talk between these two large-scale systems (see also Pessoa & Engelmann, 2010). Collectively, these results contribute to bridging the existing gap in the research on the neural effects of anxiety on specifically social processes, elucidating how incidental and dispositional anxiety interactively modulate the neural circuitry underpinning social cognition. Our findings can inform future clinical research on anxiety disorders, such as SAD, OCD, and PTSD, which often show reduced mentalizing abilities. By providing insights into the complex interplay between anxiety and its impact on multiple networks that support mentalizing, we hope to enable a way for developing more targeted and neurobiologically informed treatment approaches.

## Supplementary Material

Supplementary Material

## Data Availability

Behavioral Data, Analysis Code, and T-Maps are available at OSF (https://osf.io/8cdv7/files/osfstorage). Additional data will be made available upon request.
